# Supplementary material for: Citywide serosurveillance of the initial SARS-CoV-2 outbreak in San Francisco
Source: Res Sq. 2021 Feb 4:rs.3.rs-180966. Preprint. [Version 1] doi: 10.21203/rs.3.rs-180966/v1 (PMC7872360; doi:10.21203/rs.3.rs-180966/v1)
Supplement: Supplementary file 1 [file fc63424a18043a6484397a60.pdf]

Supplementary Information: "*Citywide serosurveillance of the initial SARS-CoV-2 outbreak in San Francisco*"

Isobel Routledge, Adrienne Epstein, Saki Takahashi *et al.*

Contents

Supplementary Table 1: Estimates of test performance characteristics from the overall procedure.

Supplementary Table 2: Seroprevalence stratified by demographic group

Supplementary Table 3: Seroprevalence stratified by neighborhood

Supplementary Text 1: Supplementary Methods

Supplementary Table 1: *Estimates of test performance characteristics from the overall procedure.*

Parameter	Point estimate and 95% CrI
Se_ELISA	mean = 97.4%, median = 97.6%, 95% CrI = 93.9%, 99.4%
Se_Luminex	mean = 95.0%, median = 95.2%, 95% CrI = 90.9%, 98.0%
Sp_ELISA	mean = 97.5%, median = 97.8%, 95% CrI = 93.7%, 99.6%
Sp_Luminex	mean = 99.2%, median = 99.4%, 95% CrI = 97.0%, 100.0%
covariance_Se	mean = 1.3%, median = 1.0%, 95% CrI = -0.04%, 3.9%
covariance_Sp	mean = 0.4%, median = 0.2%, 95% CrI = 0.0%, 1.7%
Se_overall	mean = 93.7%, median = 94.0%, 95% CrI = 89.0%, 97.2%
Sp_overall	mean = 99.6%, median = 99.8%, 95% CrI = 98.2%, 100.0%
adjusted prevalence (overall)	mean = 4.3%, median = 4.2%, 95% CrI = 2.1%, 6.3%

Supplementary Table 2: Seroprevalence stratified by demographic group

Table showing raw seroprevalence and point estimates and 95% credible intervals for posterior estimates of seroprevalence adjusted for test performance by demographic group.

Class	Group	Positive	Count	Seroprevalence	Adjusted seroprevalence	2.5% Credible Interval	97.5% Credible Interval
Sex	F	72	2491	0.0289	0.0270	0.0109	0.0361
Sex	M	119	2231	0.0533	0.0531	0.0373	0.0661
Age Group	0-19	11	281	0.0391	0.0410	0.0160	0.0690
Age Group	20-39	51	1261	0.0404	0.0399	0.0229	0.0545
Age Group	40-59	72	1322	0.0545	0.0546	0.0364	0.0713
Age Group	60-79	41	1390	0.0295	0.0281	0.0110	0.0404
Age Group	80+	17	481	0.0353	0.0363	0.0155	0.0580
Insurance Type	Government	120	2937	0.0409	0.0398	0.0232	0.0504
Insurance Type	Private or Employer	50	1421	0.0352	0.0341	0.0157	0.0474
Insurance Type	Uninsured	16	269	0.0595	0.0634	0.0315	0.0990
Insurance Type	Unknown	6	108	0.0556	0.0643	0.0228	0.1231
Hospital Group	UCSF	119	3037	0.0392	0.0379	0.0208	0.0486
Hospital Group	ZSFG	73	1698	0.0430	0.0419	0.0244	0.0557
Race/ Ethnicity	Asian	30	1206	0.0249	0.0233	0.0079	0.0353
Race/ Ethnicity	Black or African American	28	591	0.0474	0.0483	0.0281	0.0696
Race/ Ethnicity	Hispanic	53	858	0.0618	0.0633	0.0437	0.0829
Race/ Ethnicity	Other	14	348	0.0402	0.0422	0.0202	0.0691
Race/ Ethnicity	White	65	1675	0.0388	0.0379	0.0230	0.0506
Month	March	6	192	0.0313	0.0341	0.0097	0.0655
Month	April	44	959	0.0459	0.0459	0.0274	0.0631
Month	May	85	2047	0.0415	0.0404	0.0240	0.0529
Month	June	56	1526	0.0367	0.0358	0.0189	0.0490

Supplementary Table 3: Seroprevalence stratified by neighborhood

Table showing raw seroprevalence and point estimates and 95% credible intervals for posterior estimates of seroprevalence adjusted for test performance by neighborhood and for individuals experiencing homelessness. Adjusted seroprevalence was not estimated for neighborhoods with sample sizes below 50 samples from unique individuals.

Neighborhood	Positive	Count	Seroprevalence	Adjusted seroprevalence	2.5% Credible Interval	97.5% Credible Interval
Bayview	15	206	0.0728	0.0809	0.0456	0.1232
Bernal Heights	2	103	0.0194	0.0287	0.0040	0.0734
Castro/Upper Market	3	102	0.0294	0.0393	0.0089	0.0826
Chinatown	2	42	0.0476	NA	NA	NA
Crocker Amazon	4	49	0.0816	NA	NA	NA
Diamond Heights	1	14	0.0714	NA	NA	NA
Downtown/Civic Center	14	277	0.0505	0.0571	0.0305	0.0900
Excelsior	15	200	0.0750	0.0834	0.0450	0.1280
Financial District	1	51	0.0196	0.0387	0.0023	0.1053
Glen Park	3	37	0.0811	NA	NA	NA
Golden Gate Park	0	1	0.0000	NA	NA	NA
Haight Ashbury	2	95	0.0211	0.0310	0.0034	0.0796
Inner Richmond	4	144	0.0278	0.0340	0.0089	0.0721
Inner Sunset	8	157	0.0510	0.0580	0.0240	0.0988
Lakeshore	3	105	0.0286	0.0383	0.0095	0.0857
Marina	1	63	0.0159	0.0309	0.0019	0.0902
Mission	11	342	0.0322	0.0327	0.0139	0.0550
Nob Hill	1	80	0.0125	0.0245	0.0016	0.0685
Noe Valley	2	115	0.0174	0.0256	0.0028	0.0656
North Beach	4	80	0.0500	0.0615	0.0187	0.1282
Ocean View	5	140	0.0357	0.0431	0.0125	0.0841
Outer Mission	5	127	0.0394	0.0469	0.0160	0.0875
Outer Richmond	7	166	0.0422	0.0485	0.0192	0.0919
Outer Sunset	8	273	0.0293	0.0326	0.0121	0.0574
Pacific Heights	6	89	0.0674	0.0800	0.0309	0.1471
Parkside	5	144	0.0347	0.0413	0.0138	0.0833
Potrero Hill	5	88	0.0568	0.0699	0.0243	0.1318
Presidio	0	18	0.0000	NA	NA	NA
Presidio Heights	0	39	0.0000	NA	NA	NA
Russian Hill	0	49	0.0000	NA	NA	NA
Seacliff	0	15	0.0000	NA	NA	NA
South of Market	13	346	0.0376	0.0405	0.0197	0.0654
Twin Peaks	3	46	0.0652	NA	NA	NA
Visitacion Valley	4	121	0.0331	0.0410	0.0114	0.0835
West of Twin Peaks	5	141	0.0355	0.0427	0.0144	0.0855
Western Addition	5	305	0.0164	0.0221	0.0064	0.0434
Homeless	16	157	0.1019	0.1078	0.0614	0.1652

Supplementary Text 1: Supplementary Methods

Estimating test performance and positivity cutoffs for the serological assays

Selecting samples for confirmatory testing: All 5,244 SCALE-IT laboratory samples (corresponding to 4,735 unique patients) were first screened on the ELISA platform. In addition, 117 positive control samples from the LIINC cohort and 93 negative control samples were tested on this platform. The antibody concentration of each sample was calculated from the ELISA OD value using a plate-specific standard curve from serial dilutions of a pool of positive control samples. Based on the distributions of concentration values among these control samples, SCALE-IT samples with a concentration value above 0.049 were selected for confirmatory testing, corresponding to test performance characteristics of 98.3% sensitivity and 97.8% specificity.

Determining seropositivity of SCALE-IT samples: Based on the above, 653 SCALE-IT samples were selected for confirmatory testing on the Luminex platform, on which we included three SARS-CoV-2 antigens (one preparation each of the S, RBD, and N proteins). In addition, 260 positive control samples from the LIINC cohort and 114 negative control samples were tested on this platform. The antibody concentration of each antigen of each sample was calculated from the Luminex MFI value using a plate-specific standard curve from serial dilutions of a pool of positive control samples.

We then fit a multiple logistic regression model to the control samples and their Luminex concentration values for the three antigens. We used this model to classify each SCALE-IT sample as seropositive or seronegative; samples with a predicted probability value which corresponded to a specificity of 100.0% and sensitivity of 95.8% (AUC: 0.983) were classified as seropositive. The five-fold cross-validated sensitivity of this algorithm, fixing specificity at 100.0%, was estimated to be 95.4%. Given the relatively low expected seropositivity in the population, we chose to maximize the specificity of this classifier.

Determining the test performance characteristics of the two-assay procedure: The test performance characteristics of a single assay (i.e., sensitivity and specificity) can be determined from a 2x2 table of positive/negative control samples and their binary classification on that assay using a binomial model[1]. For a two-assay scenario, the binomial model can be extended to a multinomial framework where each control sample has two test results: their binary classification on each of the two assays[2]. Importantly, there may be conditional dependence between assays, where conditional on the true disease status of a given sample, the test performance of one assay may vary depending on the result on the other assay. The magnitude of this conditional dependence between two assays can be directly estimated based on the results of control samples that have been tested on both assays.

Here, of our 266 unique positive control samples, 111 were tested on both platforms (108 classified as positive by both, 1 classified as positive by ELISA and negative by Luminex, and 2 classified as negative by both), 149 were tested only on Luminex (141 classified as positive, 8 classified as negative), and 6 were tested only on ELISA (all 6 classified as positive). Of our 119 unique negative control samples, 88 were tested on both platforms (87 classified as negative by both, 1 classified as positive by ELISA and negative by Luminex), 26 were tested only on Luminex (all 26 classified as negative), and 5 were tested only on ELISA (4 classified as negative, 1 classified as positive).

We employed a modeling framework that jointly estimates assay-specific sensitivities (*Se_ELISA* and *Se_Luminex*), assay-specific specificities (*Sp_ELISA* and *Sp_Luminex*), correlation between sensitivities (*covariance_Se*), correlation between specificities (covariance_Sp), and seroprevalence. We allowed control samples that were tested only on one assay to contribute to the estimation of that assay's performance characteristics using the standard binomial model.

As the SCALE-IT samples were tested in a serial procedure that required a sample to be positive on the two assays to be classified as seropositive, we estimated the overall sensitivity of the approach as: *Se_overall* = *Se_ELISA* * *Se_Luminex* - *covariance_Se*, and the overall specificity of the approach as: *Sp_overall* = 1-(1-*Sp_ELISA*)*(1-*Sp_Luminex*) - *covariance_Sp*. Using these estimates of overall sensitivity and specificity, we obtained adjusted estimates of seroprevalence as: *adjusted prevalence* = (*raw prevalence* + *Sp_overall* - 1) / (*Se_overall* + *Sp_overall* - 1)[1]. The posterior estimates of these parameters are provided in Supplementary Table 1. The code to implement this model is included in our GitHub repository.

Calculating under-ascertainment

We compared our seroprevalence estimates to the weighted average of weekly cumulative incidence of reported cases up until June 14 2020 from the San Francisco Department of Public Health[3] and using the estimated population size of 881,549 in San Francisco according to the 2019 American Community Survey[4]. To estimate the proportion of overall infections that are ascertained, we weighted the weekly cumulative case counts by the proportion of our sample sampled in the corresponding week, lagged by two weeks collection to reflect the approximate time to sero-conversion among newly infected individuals[5].

Supplement References

1. Rogan, W. J., & Gladen, B. Estimating prevalence from the results of a screening test. *American journal of epidemiology*, **107(1)**, 71–76 (1978). ://doi.org/10.1093/oxfordjournals.aje.a112510

2. Gardner, I. A., Stryhn, H., Lind, P. & Collins, M. T. Conditional dependence between tests affects the diagnosis and surveillance of animal diseases. *Prev. Vet. Med.* **45**, 107–122 (2000).

3. [Data] City and County of San Francisco. *COVID-19 Cases and Deaths*. Available at: https://data.sfgov.org/stories/s/dak2-gvuj.

4. [Data] United States Census Bureau. *American Community Survey Data Releases.* Available at: https://www.census.gov/programs-surveys/acs/news/data-releases.html.

5. Long, Q.-X. *et al.* Antibody responses to SARS-CoV-2 in patients with COVID-19. *Nat. Med.* **26**, 845–848 (2020).
